# Investigating the effect of hypertension on vascular cognitive impairment by using the resting-state functional connectome

**DOI:** 10.1038/s41598-024-54996-9

**Published:** 2024-02-25

**Authors:** Tai-Hsin Hung, Vincent Chin-Hung Chen, Yu-Chen Chuang, Yen-Hsuan Hsu, Wen-Chau Wu, Yuan-Hsiung Tsai, Roger S. McIntyre, Jun-Cheng Weng

**Affiliations:** 1 School of Medicine, Chang Gung University, Taoyuan, Taiwan; 2 Department of Psychiatry, Chang Gung Memorial Hospital, Chiayi, Taiwan; 3 Institute of Medical Device and Imaging, Graduate Institute of Clinical Medicine, National Taiwan University, Taipei, Taiwan; 4 Department of Medical Imaging, National Taiwan University Hospital, Taipei, Taiwan; 5 Department of Psychology, National Chung Cheng University, Chiayi, Taiwan; 6 Department of Diagnostic Radiology, Chang Gung Memorial Hospital, Chiayi, Taiwan; 7 Mood Disorder Psychopharmacology Unit, Department of Psychiatry, University Health Network, University of Toronto, Toronto, ON Canada; 8 Institute of Medical Science, University of Toronto, Toronto, ON Canada; 9 Departments of Psychiatry and Pharmacology, University of Toronto, Toronto, ON Canada; 10 Department of Medical Imaging and Radiological Sciences, Chang Gung University, No. 259, Wenhua 1st Rd., Guishan Dist., Taoyuan, 33302 Taiwan; 11 Department of Artificial Intelligence, Chang Gung University, Taoyuan, Taiwan

**Keywords:** Resting-state functional MRI, Hypertension, Vascular cognitive impairment, Executive dysfunction, Functional connectivity, Neurodegeneration, Hypertension

## Abstract

Hypertension (HTN) affects over 1.2 billion individuals worldwide and is defined as systolic blood pressure (BP) ≥ 140 mmHg and diastolic BP ≥ 90 mmHg. Hypertension is also considered a high risk factor for cerebrovascular diseases, which may lead to vascular cognitive impairment (VCI). VCI is associated with executive dysfunction and is also a transitional stage between hypertension and vascular dementia. Hence, it is essential to establish a reliable approach to diagnosing the severity of VCI. In 28 HTN (51–83 yrs; 18 males, 10 females) and 28 healthy controls (HC) (51–75 yrs; 7 males, 21 females), we investigated which regions demonstrate alterations in the resting-state functional connectome due to vascular cognitive impairment in HTN by using the amplitude of the low-frequency fluctuations (ALFF), regional homogeneity (ReHo), graph theoretical analysis (GTA), and network-based statistic (NBS) methods. In the group comparison between ALFF/ReHo, HTN showed reduced spontaneous activity in the regions corresponding to vascular or metabolic dysfunction and enhanced brain activity, mainly in the primary somatosensory cortex and prefrontal areas. We also observed cognitive dysfunction in HTN, such as executive function, processing speed, and memory. Both the GTA and NBS analyses indicated that the HTN demonstrated complex local segregation, worse global integration, and weak functional connectivity. Our findings show that resting-state functional connectivity was altered, particularly in the frontal and parietal regions, by hypertensive individuals with potential vascular cognitive impairment.

## Introduction

Hypertension, a major cause of premature death, has become the most critical and largest public health problem. It affects approximately 1.2 billion individuals worldwide^1^. Hypertension was estimated to have occurred in 31.1% of adults globally in 2010^2^ and 23.5% in 2002 in Taiwan^3^, defined as systolic blood pressure (BP) ≥ 140 mmHg and diastolic BP ≥ 90 mmHg. Hypertension is also considered a high risk factor for cerebrovascular disease (CVD), stroke, cerebral small vessel disease (CSVD), and dementia^4–7^. Furthermore, recent findings point out that the brain is an early target of hypertension-induced organ damage. Such damage may affect the brain’s structure and function, modified by potential factors such as aging, chronic hypertension, and antihypertensive medication use^8^.

Previously, magnetic resonance imaging (MRI) was used to study neuroanatomy and determine how the brain functions and changes with hypertension disease^9,10^. Morphologically, the volume of the prefrontal cortex is smaller, with pathological frontal white matter changes^9,10^. This is consistent with results that show hypertension-related cognitive impairment mainly involves frontal-related executive function^11,12^. However, studies that use conventional MRI give inconsistent results and show only a moderate correlation between white matter lesions and cognitive impairment^13^.

The disruption of neurovascular coupling response might contribute to HTN-related impairment of brain activity. Hypertension may disrupt this delicate balance of neurovascular coupling in several ways, including vascular dysfunction, autoregulation impairment, endothelial dysfunction, and chronic hypoperfusion. If hypertension is associated with disruptions in neurovascular coupling, it can have a direct impact on functional connectivity in the brain. Functional connectivity refers to the synchronized activity between different brain regions, and it is crucial for various cognitive functions. When neurovascular coupling is impaired, it can lead to altered patterns of functional connectivity in the brain. Some regions may become less synchronized or less efficiently connected due to differences in blood flow and metabolic support^14^.

Functional magnetic resonance imaging (fMRI) is rapidly emerging as a tool to reliably assess the relationship between neural activity and whole-brain processing. In addition, functional resting-state MRI (rs-fMRI) is a common approach to brain imaging that detects spontaneous fluctuations in blood oxygen level-dependent (BOLD) signals. The BOLD signal is dependent on changes in deoxyhemoglobin caused by local increases/decreases in cerebral blood flow (CBF) and cerebral blood oxygenation.

Zou et al. reported that the amplitude of the low-frequency fluctuations (ALFF) of the BOLD signal reflects the intensity of spontaneous neural activity without a specific task^15,16^. ALFF levels can be used to detect physiologically important brain activity. On the other hand, the regional homogeneity approach (ReHo) is defined as the synchronicity of cluster time correlations. ReHo values are calculated using Kendall’s concordance coefficient (KCC)^17^. The ALFF and ReHo approaches both indirectly assess neural markers reflecting local brain activity.

In addition to its use in investigating local brain activity, graph theoretical analysis (GTA) revealed that the properties of complex brain networks exhibit several topological parameters and quantitatively represent a tendency toward small-world networks^18,19^. Network-based statistics (NBS) can be used to investigate significantly different associations between two groups in the pairwise brain nodes^20^. By using GTA and NBS analyses, the differences in the interregional connectivity related to hypertensive individuals with cognitive impairment can be revealed.

When it comes to hypertension and its impact on cognitive function, there is a link between alterations in resting-state functional connectivity and cognitive deficits in hypertensive individuals. Hypertension can lead to disruptions in neurovascular coupling and vascular dysfunction in the brain, as mentioned earlier^14^. Cognitive functions, such as memory, attention, executive function, and information processing, rely on efficient communication and coordination between various brain regions. Changes in functional connectivity can influence the brain’s ability to perform these functions optimally. Vascular cognitive impairment (VCI) is one of many disorders that has been associated with executive dysfunction. It is caused by various types of cerebral vascular disease related to abnormal cognition, including cerebral small vascular disease and subclinical vascular brain injury^21^. Accumulating evidence has revealed that hypertension is related to cognitive change and vascular dementia. Hypertensive (HTN) patients are 2.8 times more likely to have VCI 4 years after diagnosis of HTN, which is associated with vascular factors. HTN patients also have a greater risk of developing vascular dementia^22^. Patients with untreated HTN are 4.3 times more vulnerable to VCI^23^. Fortunately, the risk factors for vascular cognitive impairment can be prevented, postponed, and treated. Hence, there is an essential need for more research because any further decline in cognition might be preventable in the early stages of VCI^24^. The evidence also revealed that hypertensive patients had worse executive and memory/learning performance than normal controls^25^.

Based on the above, we hypothesized that resting-state functional connectivity might be altered, particularly in the frontal and parietal regions, by vascular cognitive impairment in hypertensive (HTN) individuals. Hence, we investigated whether these regions show alterations in the resting-state functional connectome due to vascular cognitive impairment in HTN by using the mean fractional ALFF (mfALFF), mean ReHo (mReHo), GTA, and NBS methods. In addition, the association between cognitive impairment and mfALFF/mReHo was also assessed.

## Results

### Demographics evaluation

We divided the 56 participants into two groups, the HTN group and the HC group. Their detailed demographic and clinical characteristics are summarized in Table 1. The two groups showed significant differences in sex and DSS score, with *p* value < 0.05. Then, sex, age, and years of education were used as covariates.

**Table 1:** Summary of the demographic and clinical characteristics.

	HTN (n = 28)		HC (n = 28)		*p* value
	Mean	SD	Mean	SD	
Males, females	18, 10	N/A	7, 21	N/A	0.002
Age (years)	61.75	7.01	60.14	7.18	0.61
Years of education	10.61	3.79	11.04	3.27	0.40
CVVLT	24.04	5.39	26.53	3.38	0.04
DSS	53.93	17.85	62.93	15.05	0.04
TMT	46.05	46.05	42.93	24.20	0.13
CGUOFT	21.97	21.97	15.46	10.78	0.27
Systolic blood pressure (mmHg)	144.67	15.75	125.77	14.10	< 0.001
Diastolic blood pressure (mmHg)	86.29	9.74	72.17	12.19	< 0.001
Duration of hypertension (years)	9.00	7.09			

*HC* healthy controls, *SD* standard deviation, *CVVLT* Chinese version verbal learning test, *N/A* not applicable, *DSS* WAIS-III digit symbol substitution, *TMT* trail making test, and *CGUOFT* Chang Gung University Orthographical Fluency Test.

### Group comparison of mfALFF and mReHo

**Figure 1:**
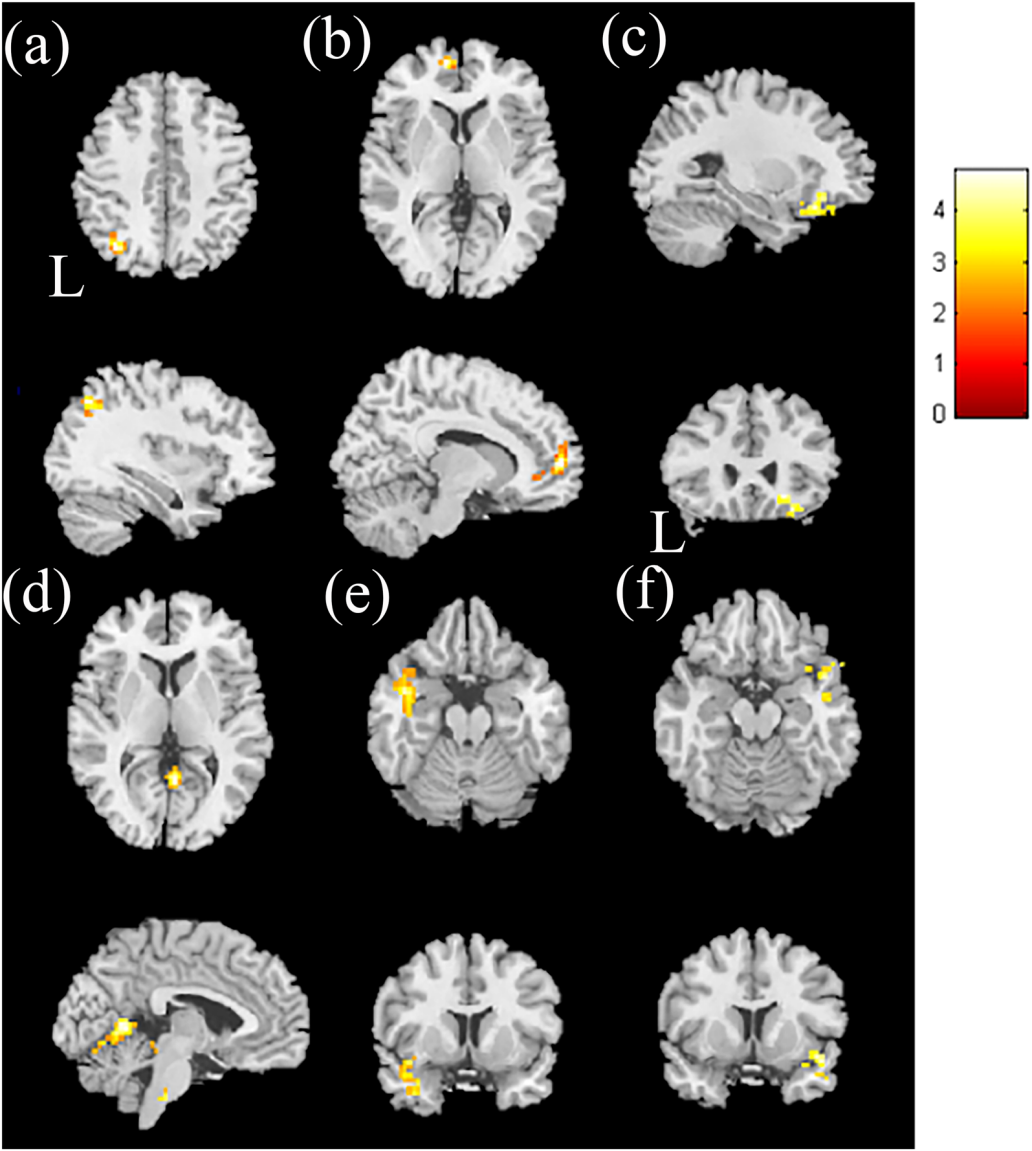
The two-sample t-test results of higher mfALFF in hypertensive patients compared with healthy controls. Higher mfALFF was found in the (**a**) left superior parietal lobule, (**b**) left medial frontal gyrus, and (**c**) right inferior frontal gyrus in the HTN group than in the HC group, with corrected *p* < 0.02 and cluster size > 50. The two-sample t-test results of lower mfALFF in hypertensive patients compared with healthy controls. Lower mfALFF was found in the HTN group than in the HC group in the (**d**) right posterior cingulate gyrus, (**e**) left superior temporal gyrus, and (**f**) right superior temporal gyrus, with *p* < 0.02 and cluster size > 100. The color bar indicates the t-score.

In the group comparison between HTN patients and HC in the mfALFF, the results indicated that in the HTN patients, significantly higher activity was found in the left superior parietal lobule, left medial frontal gyrus, and right inferior frontal gyrus, with a corrected *p* value less than 0.02. The HTN patients showed significantly lower activation than HC in the right posterior cingulate gyrus and bilateral superior temporal gyrus, with a corrected *p* value less than 0.02 (Fig. 1 and Table S1).

**Figure 2:**
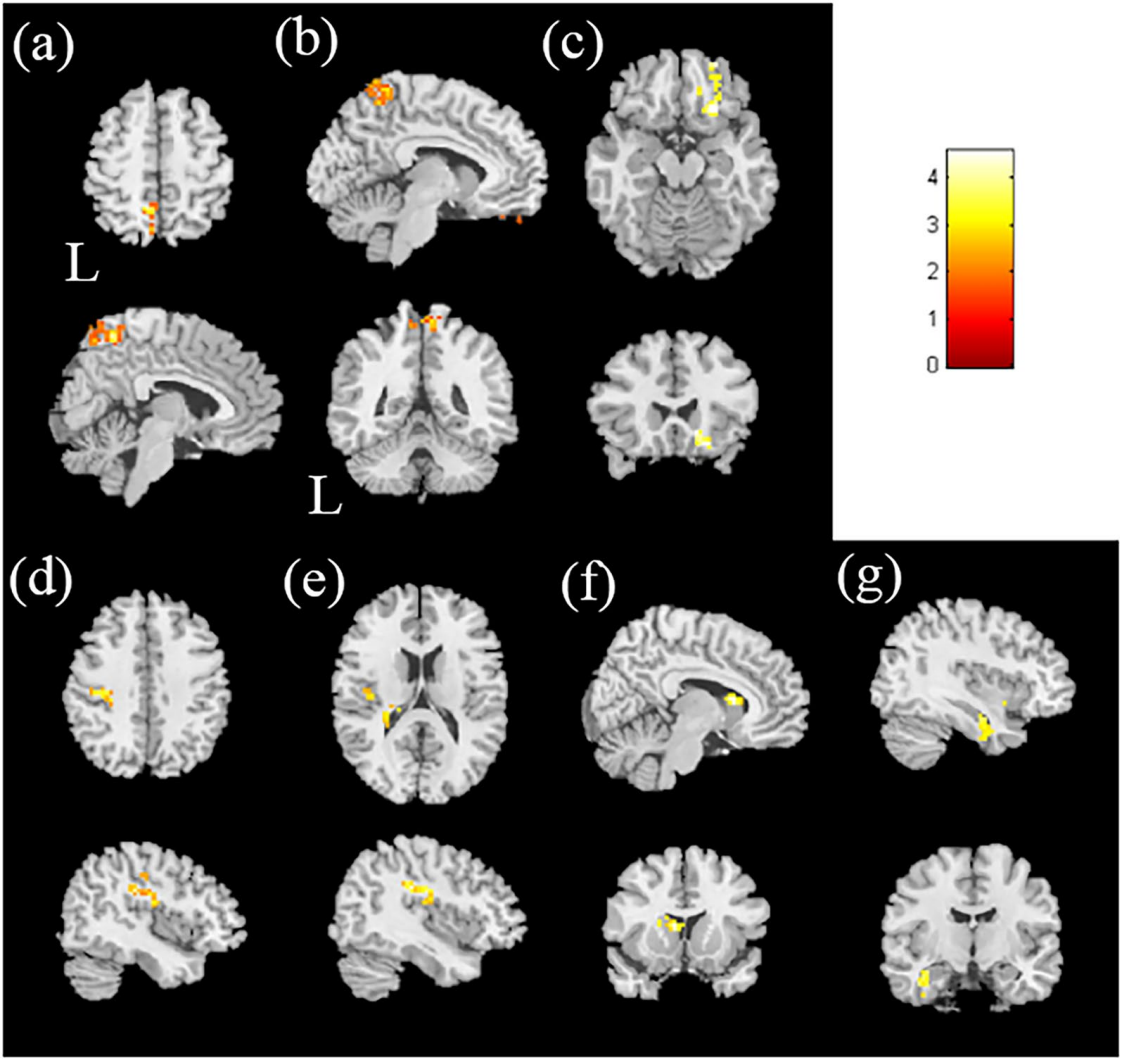
The two-sample t-test results of mReHo in hypertensive patients compared with healthy controls. Higher mReHo was found in the (**a**) left parietal lobule, (**b**) right postcentral gyrus, and (**c**) right inferior frontal gyrus in the HTN group than in the HC group, with corrected *p* < 0.02 and cluster size > 100. The two-sample t-test results of mReHo in hypertensive patients compared with healthy controls. Lower mReHo was found in the HTN group than in the HC group in the (**d**) left precentral gyrus, (**e**) left insula, (**f**) left caudate, and (**g**) left fusiform gyrus, with *p* < 0.02 and cluster size > 50. The color bar indicates the t-score.

In the analysis of mReHo between the HTN patients and HC, we found higher mReHo values in the left parietal lobule, right postcentral gyrus, and right inferior frontal gyrus in the HTN patients, with a corrected *p* value < 0.02 compared with HC. We also found lower mReHo values in the left precentral gyrus, left insula, left caudate, and left fusiform gyrus in the HTN patients, with a corrected *p* value < 0.02 (Fig. 2 and Table S2).

### Association between CVVLT and mfALFF/mReHo

In the 28 HTN participants’ multiple regression analysis of the correlation between the CVVLT and mfALFF, we found a positive correlation with the right superior frontal gyrus, left precentral gyrus, and right postcentral gyrus, with a corrected *p* value < 0.02, and no negative correlation was found.

**Figure 3:**
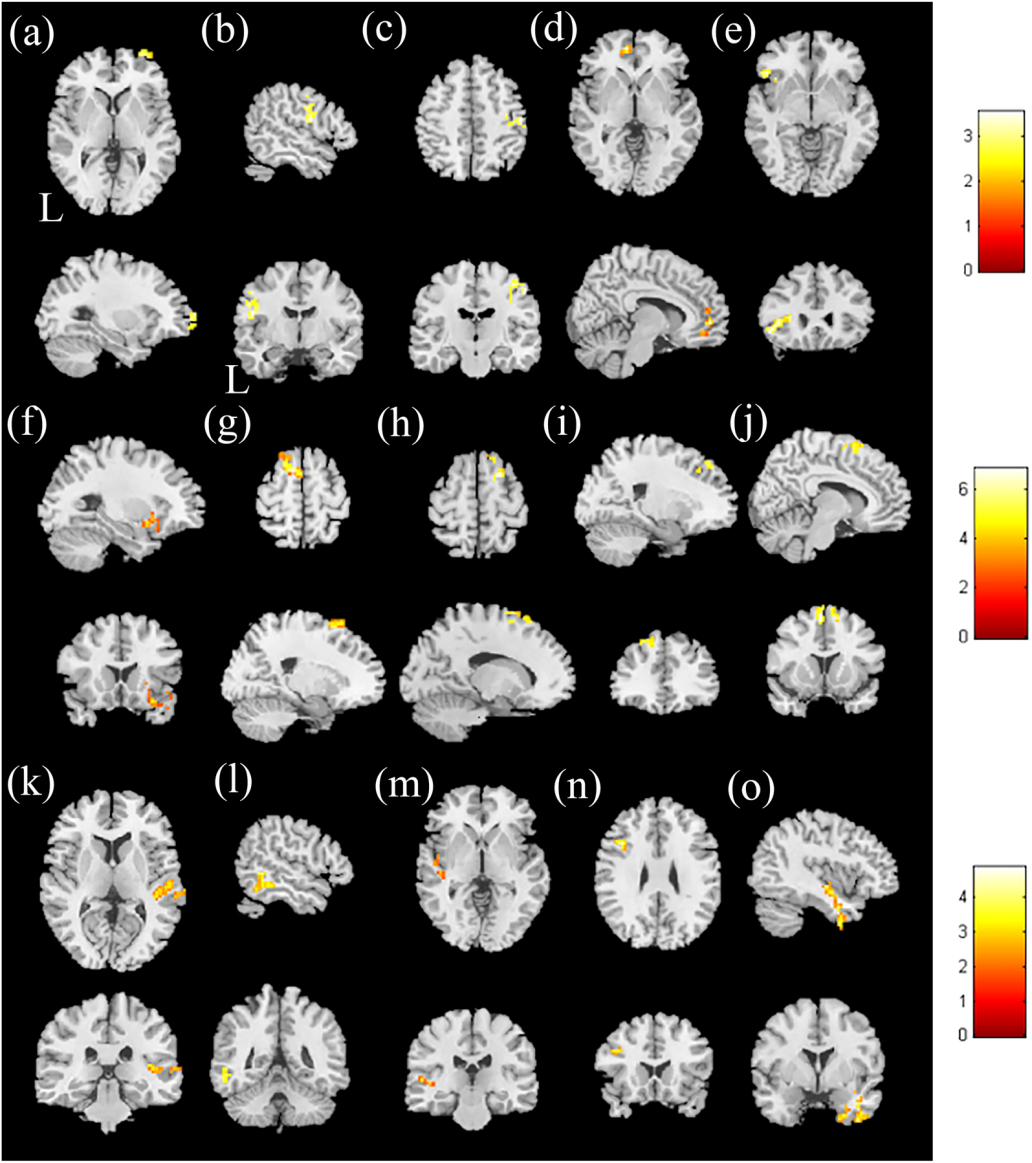
Correlation between CVVLT and mfALFF in the HTN group. The t-score of t-statistics and scatter plot indicate a positive correlation in the (**a**) right superior frontal gyrus, (**b**) left precentral gyrus, and (**c**) right postcentral gyrus. The correlation between the CVVLT and mReHo in the HTN group. The t-score of the t-statistics and scatter plot indicate a positive correlation in the (**d**) left medial frontal gyrus and (**e**) left inferior frontal gyrus, with *p* < 0.02 and cluster size > 40. The correlation between TMT and mfALFF in the HTN group. The t-score of t-statistics and scatter plot indicate a positive correlation in the (**f**) right inferior frontal gyrus; a negative correlation was found in the (**g**) left middle frontal gyrus and (**h**) right superior frontal gyrus. Correlation between TMT and mReHo scores in the HTN group. The t-score of t-statistics and scatter plot indicate a negative correlation in the (**i**) left middle frontal gyrus and (**j**) left superior frontal gyrus, with *p* < 0.02 and cluster size > 50. Correlation between CGUOFT and mfALFF in the HTN group. The t-score of t-statistics and scatter plot indicate a positive correlation in the (**k**) right superior temporal gyrus and (**l**) left inferior temporal gyrus. Correlation between CGUOFT and mReHo in the HTN group. The t-score of t-statistics and scatter plot indicate a positive correlation in the (**m**) left insula, (**n**) left middle frontal gyrus, and (**o**) right inferior temporal gyrus, with *p* < 0.02 and cluster size > 50. The color bar indicates the t-score.

In the correlation between the CVVLT and mReHo, a positive correlation was found with the left medial frontal gyrus and left inferior frontal gyrus, with a corrected *p* value < 0.02; however, no negative correlation was found (Fig. 3a–e and Table S3).

### Association between TMT and mfALFF/mReHo

As shown in Fig. 3f–h and Table S4, a multiple regression analysis of mfALFF was conducted with the Trail Making Test (TMT) score. A higher TMT score represented worse cognitive performance. The results revealed a significant positive correlation between TMT and mfALFF in the right inferior frontal gyrus, with a corrected *p* value < 0.02. A negative correlation was found in the left middle frontal gyrus and the right superior frontal gyrus between TMT and mfALFF, with a corrected *p* value < 0.02.

Figure 3i, j and Table S4 show the significant negative correlation between the TMT and mReHo scores in the left middle frontal gyrus and the left superior frontal gyrus, with a corrected *p* value < 0.04. No significant positive correlation was found between TMT and mReHo.

### Association between CGUOFT and mfALFF/mReHo

In the multiple regression analysis, a significant positive correlation between Chang Gung University Orthographical Fluency Test (CGUOFT) and mfALFF was found in the right superior temporal gyrus and left inferior temporal gyrus, with a corrected *p* value < 0.02. There was no significant negative correlation between CGUOFT and mfALFF. In the association between the CGUOFT and mReHo, a significant positive correlation between CGUOFT and mfALFF was found in the left insula, left middle frontal gyrus, and right inferior temporal gyrus, with a corrected *p* value < 0.02 (Fig. 3k–o and Table S5).

### Association between DSS and mfALFF/mReHo

**Figure 4:**
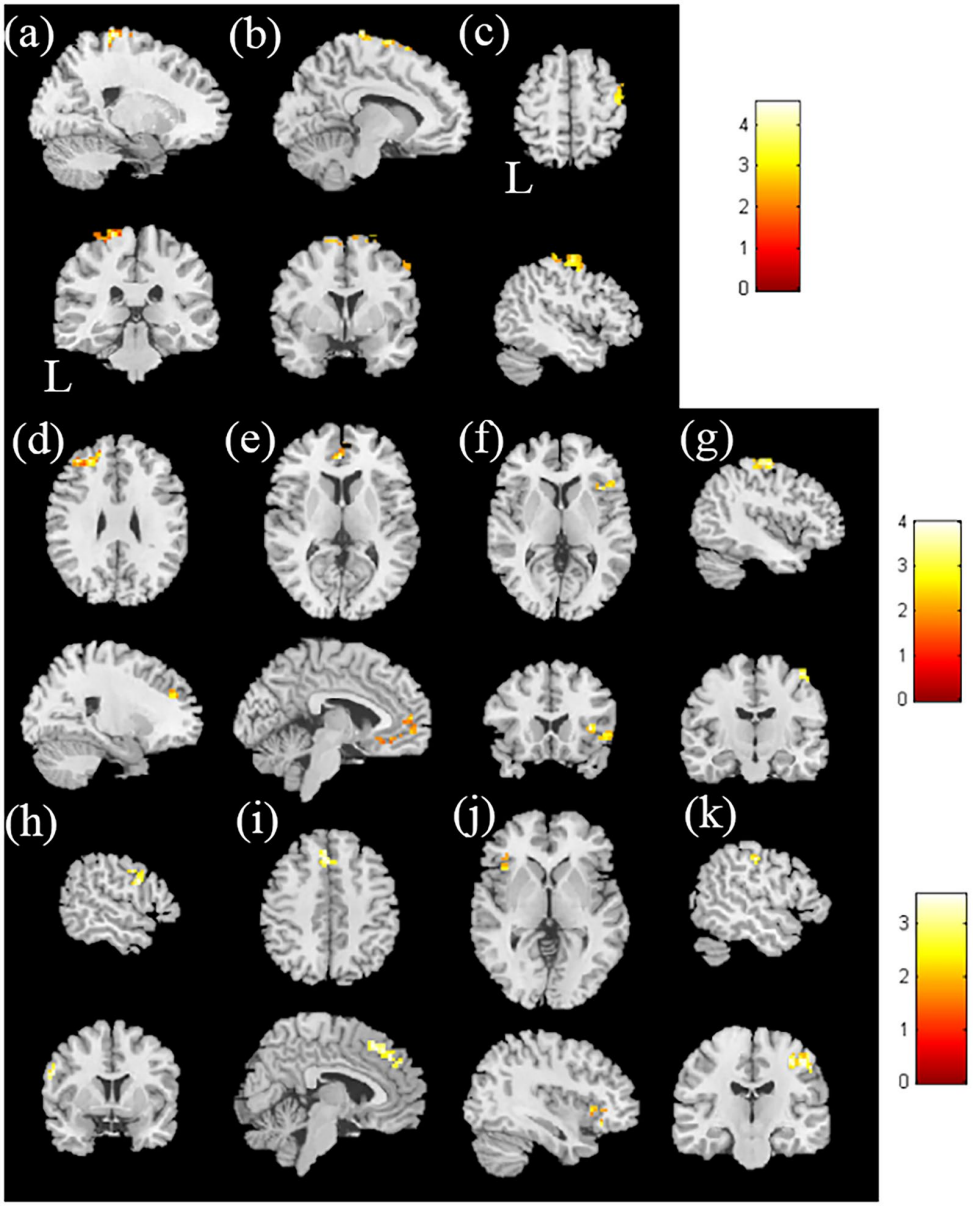
The correlation between DSS and mfALFF in the HTN group. The t-score of t-statistics and scatter plot indicate a positive correlation in the (**a**) left superior frontal gyrus, (**b**) right superior frontal gyrus, and (**c**) right middle frontal gyrus. The correlation between DSS and mReHo in the HTN group. The t-score of t-statistics and scatter plot indicate a positive correlation in the (**d**) left superior frontal gyrus, (**e**) right medial frontal gyrus, (**f**) right insula, and (**g**) right precentral gyrus, with *p* < 0.02 and cluster size > 60. The correlation between the duration of hypertension and mfALFF in the HTN group. The t-score of t-statistics and scatter plot indicate a positive correlation in the (**h**) left inferior frontal gyrus and (**i**) right medial frontal gyrus. Correlation between the duration of hypertension and mReHo in the HTN group. The t-score of t-statistics and scatter plot indicate a positive correlation in the (**j**) left inferior frontal gyrus and (**k**) right postcentral gyrus, with *p* < 0.02 and cluster size > 50. The color bar indicates the t-score.

In the association between the DSS and mfALFF, a positive correlation was found with the bilateral superior frontal gyrus and right middle frontal gyrus, with a *p* value < 0.02; in contrast, a negative correlation was not found. In the association between the DSS and mReHo, a positive correlation was found with the left superior frontal gyrus, right medial frontal gyrus, right insula, and right precentral gyrus, with a corrected *p* value < 0.02. There was no significant negative correlation between DSS and mReHo (Fig. 4a–g and Table S6).

### Association between the duration of hypertension and mfALFF/mReHo

Figure 4h, i and Table S7 show the association between the duration of hypertension and mfALFF. We found a significant positive correlation with the left inferior frontal gyrus and right medial frontal gyrus, with a corrected *p* value < 0.02. There was no significant negative correlation between the duration of hypertension and mfALFF.

In Fig. 4j, k and Table S7, we show the significant positive correlation with the left inferior frontal gyrus and right postcentral gyrus, with a corrected *p* value < 0.02. There was no significant negative correlation between the duration of hypertension and mReHo.

### Network-based results

**Figure 5:**
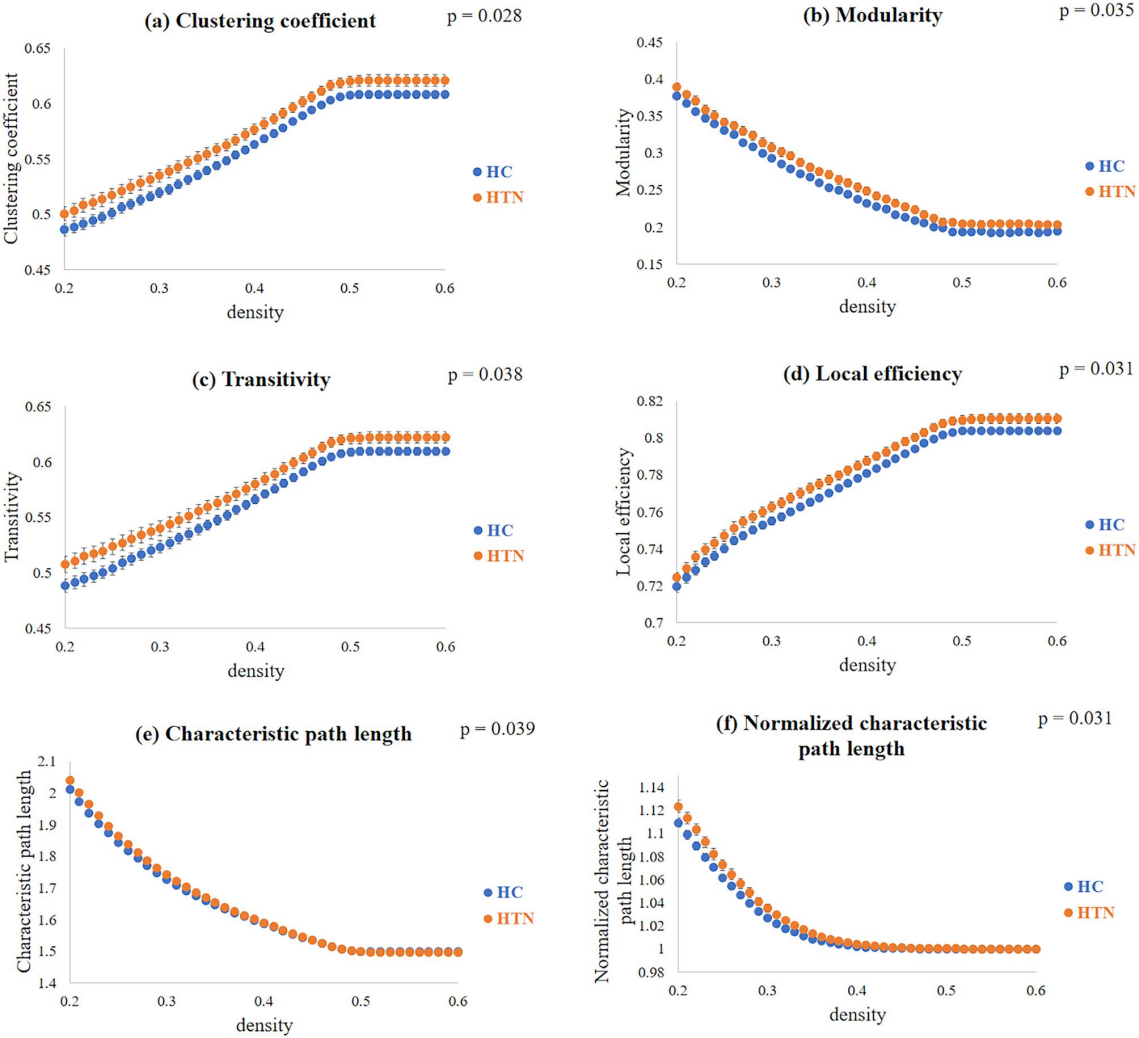
Topological parameters among the HTN and HC groups, including (**a**) clustering coefficient, (**b**) modularity, (**c**) transitivity, (**d**) local efficiency, (**e**) characteristic path length, and (**f**) normalized characteristic path length. The density indicates the fraction of current counts of edges out of all possible edges.

In the GTA, we assessed the functional connectivity between the HTN group and the HC group. We measured the area under the curve (AUC) between 0.2 and 0.6 of the density to calculate the *p* value with a two-sample t-test. Compared with the HC groups, we found significantly higher tendencies (*p* value < 0.05) in the clustering coefficient, modularity, transitivity, local efficiency, characteristic path length, and normalized characteristic path length in the HTN group (Fig. 5). However, no significant difference was found in the normalized clustering coefficient, assortativity, global efficiency, or small-worldness index between the two groups.

**Figure 6:**
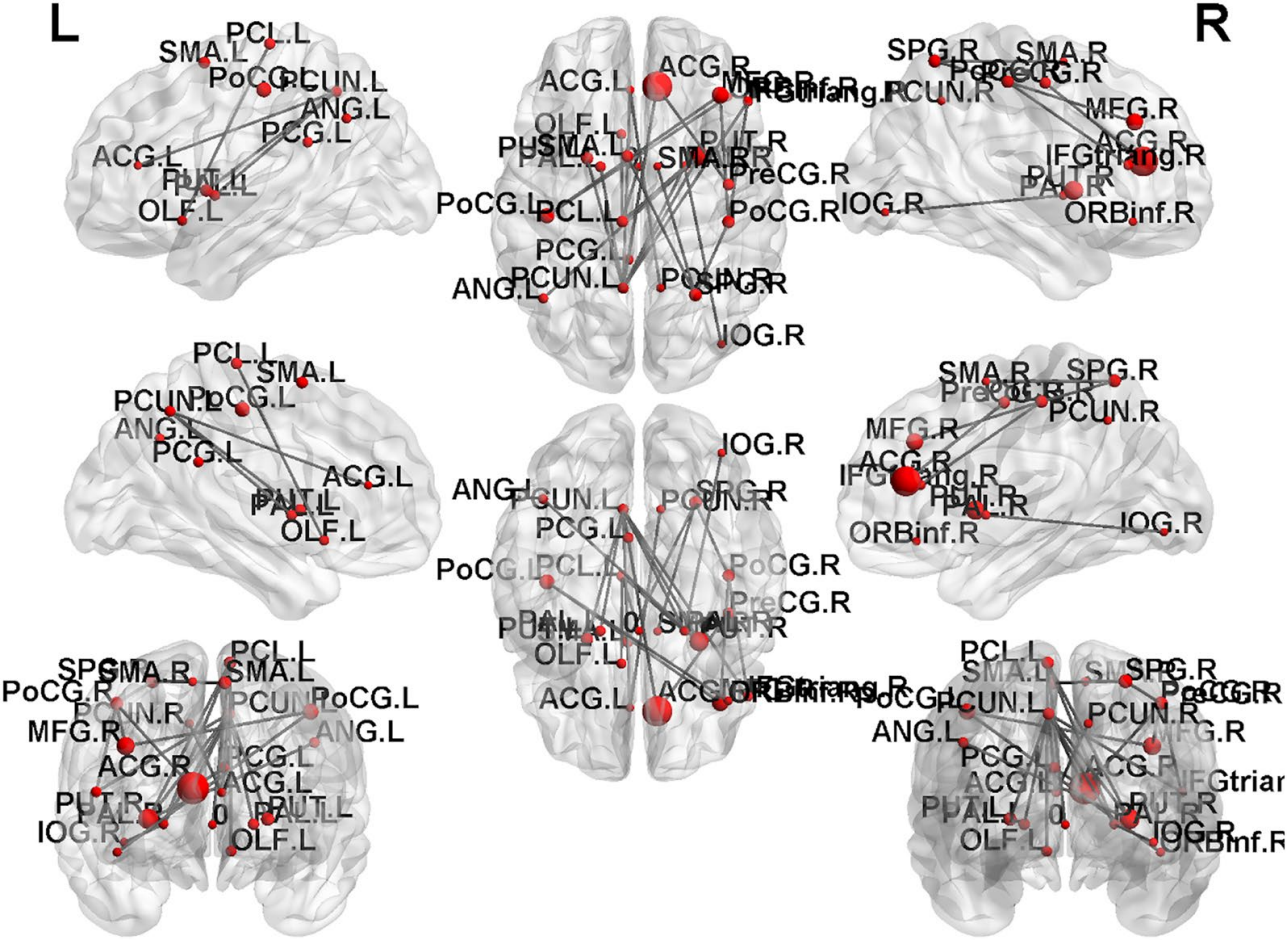
The NBS analysis showed reduced functional connectivity in the HTN group compared with the HC group (*p* value < 0.05). The disrupted subnetworks were mainly between the primary somatosensory cortex and prefrontal areas.

NBS analysis was performed to compare the edges of the functional brain networks between the HTN and HC groups. We found that the hypertensive patients had lower functional connections than the HCs in the subnetwork mainly between the primary somatosensory cortex and prefrontal areas, including from the right precentral to the right anterior cingulum, the right anterior cingulum to the left posterior cingulum, the right middle frontal gyrus to the bilateral postcentral gyrus, the right orbital portion of the inferior frontal gyrus to the left postcentral gyrus, the right triangular portion of the inferior frontal gyrus to the right postcentral gyrus, the bilateral supplementary motor area to the right superior parietal lobule, the right postcentral gyrus to the right superior parietal lobule, the right triangular portion of the inferior frontal gyrus to the left precuneus, the right anterior cingulum to the left precuneus, the left anterior cingulum to the bilateral precuneus, the left olfactory cortex to the left paracentral lobule, the right insula to the left paracentral lobule, the left precuneus to the bilateral putamen, the left angular gyrus to the right putamen, the left paracentral lobule to the right putamen, the left precuneus to the bilateral pallidum, the right inferior occipital gyrus to the right pallidum, and the left paracentral lobule to the right pallidum (*p* value < 0.05), as shown in Fig. 6.

## Discussion

### Group comparison of mfALFF and mReHo

In the present study, we observed that HTN patients had lower mfALFF values in the superior temporal gyrus and posterior cingulate, as well as lower mReHo values in the caudate, precentral gyrus, fusiform gyrus, and insula. Consistent with our results, a previous study showed CBF declines in hypertensive patients relative to controls, especially in the frontal, caudate, posterior cingulate, and superior temporal regions^26,27^. The reduction in CBF in these regions might be due to narrowing of the arteries in HTN, which decreases the blood flow and neurovascular coupling and indirectly causes decreased mfALFF signals^16^. Additionally, the decreased mReHo values might indicate the impairment of cognitive function in these regions. Reduced mReHo may reflect reduced blood flow, compromised oxygen supply, and disrupted neural synchronization, which can impair information processing and cognitive performance^14,28^. Notably, the evidence of lower metabolic brain activity and gray matter atrophy among hypertensive patients further supports a reduction in CBF. Supporting the CBF cause, Beauchet et al. indicated that higher blood pressure is generally associated with atrophy of gray matter volume, especially in the temporal regions, which is consistent with our findings^29^.

However, we found enhanced, rather than reduced, activation in hypertensive versus nonhypertensive patients, mainly in the primary somatosensory cortex and frontal cortex. Both the mfALFF and mReHo values increased in the inferior frontal gyrus. We also discovered higher mfALFF values in the medial frontal gyrus. In the parietal region, we found a higher mfALFF in the superior parietal lobule, and we also noted an increased mReHo value in the postcentral gyrus. Early work in spontaneously hypertensive rats suggested that they exhibited greater BOLD and CBF responses than normotensive controls; the activations were localized to the primary somatosensory cortex^30^. The primary somatosensory cortex is located in the postcentral gyrus, and it is responsible for receiving sensory information. Vascular abnormalities in hypertensive patients in the primary somatosensory cortex have been reported^31,32^. In addition, CBF changes were enhanced in the frontal areas among hypertensive patients relative to normal controls^33^, which is consistent with the findings that hypertension-related cognitive impairments mainly altered frontal-related executive function^11,34^. These hypertension-related changes in cerebral vessels then induced an imbalance in the regulation or redistribution of CBF, leading to cerebral vascular abnormalities and disruption of neurovascular coupling.

### Multiple regression analysis of mfALFF and mReHo

Previous studies suggested that hypertension played an important role in the development of vascular cognitive impairment (VCI), Alzheimer’s disease (AD), and vascular dementia^35^. Saxby et al. primarily investigated Caucasian-American participants and found that blood pressure was related to various cognitive processes^36^. Additionally, Suhr et al. suggested that executive function, memory, attention, verbal learning, visual tracking, immediate and delayed recall, perceptual speed, reasoning, and other cognitive domains are impacted by blood pressure^37^. As mentioned in the Materials and Methods, we conducted four cognitive tests and included the duration of hypertension in multiple regression analysis. Because different cognitive tests serve different purposes, we next discuss the breakdown of cognitive abilities in two domains: executive and memory/learning functions.

### Executive function

Executive dysfunction and information processing speed are vulnerable to hypertension. Adams et al. indicated that executive function and processing speed rely heavily on the integrity of frontal and subcortical brain structures^38^. This finding might be associated with our results, including the negative correlations between TMT and mfALFF/mReHo in the middle and superior frontal gyrus. Likewise, in the association between DSS and mfALFF, we found a positive correlation in the same region. In addition, we found a positive correlation between DSS and mReHo in the superior frontal gyrus and medial frontal gyrus. Consistent with our findings, these frontal regions might be most vulnerable to the effects of hypertension^37,39^. In a case report^40^, an untreated hypertensive man in midlife presented similar findings that the functional connectivity of the caudate to the frontal lobe was damaged, leading to executive dysfunction, personality changes, and lack of empathy. Moreover, Marshall et al. indicated that the superior temporal gyrus (STG) is a well-known damaged region in hypertensive individuals^41^. Beason-Held et al. also found abnormal changes in regional CBF within the superior temporal cortices, which were vulnerable to increased blood pressure^26^. Related to these results, we found a positive correlation between CGUOFT and mfALFF in the superior and lateral temporal gyrus. These findings also revealed an association with changes in attentional functions.

### Memory and learning function

In the present study, immediate recall total scores of the CVVLT measured the capacity to use semantic associations as a strategy for learning words in hypertensive patients. Yaffe et al. considered learning to be the ability of memory consolidation and represented the effortful activity and careful attention by the learner^42^. Previous studies have indicated that blood pressure is linked specifically to verbal learning^43^. Notably, because verbal learning is a process that remains intact well into midlife, this relationship is not reduced by age effects^43^.

Previous evidence suggested that chronically elevated blood pressure may have a considerable effect on cognitive performance, including verbal learning performance^36^. According to a large study^44^, the hypertensive group performed worse in memory than the normotensive group. This was related to our findings of a positive correlation between CVVLT and mfALFF/mReHo in the parietal and frontal regions. Similar to our observations of mfALFF, the function of the parietal lobule might be related to verbal short-term memory. In addition, Petersson et al. suggested that when verbal learning scores decreased, reductions were also observed in the frontal, parietal, and temporal cortices^45^.

### Duration of hypertension

Our results showed associations between the duration of hypertension and the mfALFF/mReHo in hypertensive patients. The positive correlation between the duration of hypertension and mfALFF in the frontal regions was related to the group comparisons in mfALFF. These findings might indicate that a longer duration causes abnormal increased cerebral blood flow. A previous study revealed positive associations between the duration of hypertension and the incidence of cognitive dysfunction and its severity^44^. This finding might be consistent with our results of a positive correlation between the duration and mReHo; that is, the localizations in the inferior frontal gyrus and postcentral gyrus might represent the major regions of cognitive dysfunction.

### Network-based analyses

Consistent with previous studies on GTA results, we found that both hypertensive patients and HCs represented a small-world state of brain functional networks^19,46^. The normalized relationship between local segregation and global integration indicated the small-world networks^47^. Small-world networks exhibit a high clustering coefficient, which means that nodes in the network tend to be connected to their neighbors, forming tightly-knit local clusters. Small-world networks also have short characteristic path lengths between nodes. This means that you can reach most nodes in the network from any other node in a relatively small number of steps^47^. In other words, if you pick a node in a small-world network and look at its neighbors, these neighbors are likely to be connected to each other as well, and distant nodes can be connected by a relatively small number of intermediate nodes, facilitating efficient information transfer.

In HTN, we found significantly higher local segregation compared with HC, including clustering coefficient, modularity, transitivity, and local efficiency. These results are in agreement with previous studies. Bullmore & Sporns revealed that comparatively overconnected brain networks were found in hypertensive patients to provide better efficiency of parallel information transfer^18^. In global integration, the characteristic path length is defined as how close on average a node in the network is connected to all other nodes in the network. According to Watts & Strogatz, path length characteristics convey information about the degree of connectivity in networks and how well information can be integrated between different systems^47^. We found a significant increase in the characteristic path length of the HTN group compared to the HC group. These observations are consistent with previous studies^48^, which showed decreased global efficiency and increased characteristic path length in hypertensive patients compared to healthy controls. While the brain of the HTN group still maintains a small world structure, there is a slight trend towards a regular network.

We compared the connectivity matrix of the brain network between the two groups (HTN and HC) using NBS and revealed significantly weakened functional connectivity in HTN. Numerous altered functional network was found within the F-statistic analysis. These nodes correspond to the following AAL regions: the anterior/posterior cingulate gyrus, middle frontal gyrus, precentral gyrus, inferior frontal gyrus, superior parietal gyrus, postcentral gyrus, precuneus, olfactory cortex, inferior occipital gyrus, putamen, pallidum, supplementary motor area (SMA), paracentral lobule, and angular gyrus.

Many studies have focused on resting-state fMRI approaches to investigate underlying dysfunctional effects in neurological and psychiatric brain diseases, such as vascular cognitive impairment (VCI) and Alzheimer’s disease (AD)^49^. Our findings are consistent with previous studies, showing that older adults with elevated blood pressure have changes in the frontoparietal control network (FCN), dorsal attention network (DAN), and ventral attention network (VAN)^50^. Due to the elevation of blood pressure, the deterioration of the functional connectivity of the VAN may be associated with alterations in attentional control focused on upward stimulation, impairing its information processing.

Furthermore, hypertension was also associated with subtle damage to white matter integrity, particularly in the corpus callosum^51^. Knyazeva et al. pointed out that the loss of functional connectivity in the spleen of the corpus callosum^52^, which is connected to the visual cortex, the posterior parietal, and cingulate regions, was closely associated with the aging process^53^ and stress tolerance^54^. In sum, previous observations indicate biological responses to stress may lead to elevated blood pressure, and our results support they may further reduce functional connectivity in the parietal and posterior cingulate regions.

### Limitations

In the present study, we conducted resting-state functional connectome analyses to investigate the effect of cognitive impairments on hypertensive patients’ brains. Functional MRI has relatively poor temporal resolution compared to other neuroimaging methods, and the BOLD signal is an indirect measure of neural activity that only reflects changes in blood oxygenation that follow changes in neural activity^55^. One should exercise caution when deducing the exchange of information between brain regions, which essentially underpins their effective connectivity, using these spatiotemporal dynamics in neuroimaging data. Therefore, multimodal approaches that combine fMRI with other imaging methods, advanced analysis methods, and experimental designs to gain a more comprehensive understanding of brain function can be developed to address some of the challenges associated with fMRI’s limitations. There are a few limitations of our study, and we suggest that these issues should be considered for further study.

First, the relatively small sample size and the difference in sex between HC and HTN groups of this study are its primary limitations. Second, Wolf-Maier et al. suggested risk factors for hypertension-related brain damage, such as ischemic/hemorrhagic stroke, severe white matter changes, obvious structural abnormalities, and territorial infarction^56^. Although we carefully excluded these underlying factors in our study, the rest of the subtle vascular dysfunction caused by normal aging in hypertension, such as small vessel disease, and the effects of antihypertensive drug use, might confound our results. The third limitation is the lack of information to quantify the severity of hypertension. We could not identify the physiological complications and their effects on brain damage between controlled and uncontrolled hypertension groups. These limitations also remained in the methods. According to a previous study^57–59^, the effects of cardiac and respiratory fluctuations were not completely filtered from a band of 0.01–0.12 Hz frequency fluctuations. Due to the aliasing effects from the cardiac and respiratory fluctuations, this noise might have reduced the specificity of the connectivity effects. In particular, Cordes et al. pointed out that sources of physiological noise have little effect on cross-correlation coefficients when defining functional connectivity maps^60^.

## Conclusions

In the present study, we conducted resting-state fMRI in hypertensive patients (HTN) and normotensive healthy controls (HC). We investigated differences in local spontaneous brain activity and global functional connectivity between the groups. In the group comparison between mfALFF/mReHo, HTN showed reduced spontaneous activity in the regions corresponding to vascular or metabolic dysfunction and enhanced brain activity, mainly in the primary somatosensory cortex and prefrontal areas. We also observed cognitive dysfunction in HTN, such as executive function, processing speed, and memory. The GTA and NBS analyses indicated that the HTN demonstrated complex local segregation, worse global integration, and weak functional connectivity. Our findings show that resting-state functional connectivity was altered, particularly in the frontal and parietal regions, by hypertensive individuals with potential vascular cognitive impairment.

## Methods

### Participants

This study was approved by the Institutional Review Board of Chang Gung Memorial Hospital, Chiayi, Taiwan (No. 201801329B0), and all methods were performed in accordance with the relevant guidelines and regulations. Written informed consent has been obtained from all participants and/or their legal guardians. In the present study, we recruited a total of 56 participants, including 28 hypertensive (HTN) patients (age 51–83 years, mean = 61.75 years, SD = 7.01 years, 18 males and 10 females) and 28 normotensive healthy controls (hereafter, “HC”) (age 51–75 years, mean = 60.14 years, SD = 7.18 years, 7 males and 21 females) from Chiayi Chang Gung Memorial Hospital. They were at least 50 years of age and right-handed. The patients with hypertension were those regularly treated in the outpatient department of the hospital. HTN patients were considered hypertensive if their blood pressure was > 140/90 mmHg^61^ at an office visit. A research assistant measured their blood pressure three times in the supine position after 5 min of rest. The three measures of systolic and diastolic blood pressure were each averaged. The exclusion criteria were a stroke history, diagnosis of major psychiatric disorders, and movement impairments.

### Cognitive tests

The cognitive tests were examined on the same day as the MRI data acquisition. These tests were selected from the list recommended by the National Institute for Neurological Disorders and Stroke (NINDS) and the Canadian Stroke Network (CSN) for VCI studies^62^, including the Digit Symbol Substitution (DSS) subtest of the Wechsler Adults Intelligence Scale—the Third Edition (WAIS-III, Wechsler, 2002), the Trail Making Test (TMT), and the Taiwanese adapted version of the California Verbal Learning Test, the Chinese Version Verbal Learning Test (CVVLT)^63^. In addition, the verbal fluency test plays an important role in evaluating executive function. Therefore, the Chang Gung University Orthographical Fluency Test (CGUOFT) was chosen as a validated evaluation. The psychological tests for all participants were administered by the researcher with a master’s degree in psychology.

The Chinese Version Verbal Learning Test (CVVLT) consists of 9 two-character nouns in a list performed over four learning trials^64^. The individuals were instructed to read the list and freely recall it immediately, and their recall was assessed. This procedure was repeated four times, and the immediate recall total scores of the Chinese Version Verbal Learning Test were calculated.

The WAIS-III Digit Symbol Substitution (DSS) has been used to forecast the susceptibility to cognitive impairment that underlies dementia, with lower scores representing worse performance^65^. The DSS score falls rapidly as dementia progresses and it shows sensitivity to brain damage. According to a smaller clinical study^66^, DSS scores are sensitive to VCI and are inversely correlated with white matter rating scores in elderly individuals.

The Trail Making Test (TMT) is separated into two parts, TMT-A and TMT-B. In each test the participant is asked to draw a line between 24 consecutive circles that are randomly arranged on a page. The TMT-A uses all numbers, whereas the TMT-B alternates numbers and letters, requiring the patient to switch between numbers and letters in consecutive order. The TMT score assesses the amount of time that an individual requires to complete a task. We asked each individual to complete the questionnaire quickly and accurately. The individual was instructed to return to the state where the error originated and then continue. TMT provides information on executive functions, mental flexibility, speed of processing, scanning, and visual search^67^.

The character fluency test has been adopted as an alternative version based on the composition of Chinese characters from the original phonemic fluency task^68^. The Chang Gung University Orthographical Fluency Test (CGUOFT) is a fluency task with Chinese characters that has good internal consistency, acceptable validity, and good test–retest reliability. Within a 4-year longitudinal study, de Menezes et al. showed that both hypertension and prehypertension coincided with a steep decline in verbal fluency^69^.

### Duration of hypertension

Accumulating evidence suggests a strong link between midlife or late-life hypertension and how severe cognitive impairment is. These findings support the hypothesis that the onset of hypertension in midlife may contribute to a higher risk of cognitive deficits in later life^35^. However, the relationship between the duration of hypertension and cognitive impairment is not fully understood. Swan et al. indicated that the duration of hypertension is linked to worse cognitive function^70^. In this case, we measured the duration of hypertension as an index to determine the link between the duration of hypertension and cognitive function.

### MRI data acquisition

All 56 participants were asked to undergo scanning in a 3-Tesla MRI system (Siemens Tim Trio scanner) with a standard 32-channel head coil. The T2* weighted echo-planar imaging (EPI) sequence was performed on the resting-state functional images, with the following parameters: TR/TE = 2000 ms/30 ms, FOV = 220 mm × 220 mm, matrix size = 64 mm × 64 mm, voxel size = 3.4 mm × 3.4 mm × 4 mm, number of slices = 31, flip angle (FA) = 90°. Each resting-state fMRI run contained 300 image volumes, and the total scan time was approximately 10 min. During the resting-state fMRI scanning, all subjects were instructed to lie in the scanner and keep their eyes closed. We also asked the participants not to sleep and not to focus their thoughts on anything in particular.

### Image preprocessing of resting-state functional MRI

We conducted functional image preprocessing using Statistical Parametric Mapping (SPM12, Wellcome Department of Cognitive Neurology, London, UK) software in MATLAB. The initial ten EPI volumes were discarded in order to ensure the stability of functional signal intensity. The imaging preprocessing procedure was mainly divided into four parts: slice-timing correlation, motion correlation, normalization, and smoothing. First, because slices cannot be acquired simultaneously in fMRI acquisition protocols, slice-timing correlation was conducted to correct for these slice-dependent temporal delays. Second, images were realigned to the first volume for the head motion correlation using affine transform. When the translation change was > 1 mm or the change in trajectory was > 1°, the images were excluded. Third, the realigned images were normalized to the standard Montreal Neurological Institute space, the East Asian template, with affine transformation. Finally, we applied a Gaussian kernel with a 6-mm full width at half-maximum (FWHM) to all images to achieve a better signal-to-noise ratio (SNR) gain. Note that smoothing was not essential for the later mReHo calculations because smoothing already has an autocorrelation.

After functional image preprocessing, we performed both linear detrending and bandpass temporal filtering by the Resting-State Data Analysis tool kit v1.8 (REST v1.8). Based on the in-built fast Fourier transform functions, REST converted time-series data (time domain) to the frequency domain and calculated the power spectrum. Tong et al. indicated that the BOLD signal results from neuronal signals with slow hemodynamic reactions^71^. Josephs et al. indicated that the frequencies of neuronal BOLD signals are almost below 0.15 Hz^72^. Therefore, Cordes et al. demonstrated that important physiological significance remains after filtering the higher-frequency components by low-frequency fluctuations (from 0.01 to 0.08 Hz)^60^. On the other hand, Baria et al. observed that brain networks also operated in high-frequency fluctuations from 0.1 to 0.12 Hz^73^. Eventually, we investigated the complex functional networks at frequencies ranging from 0.01 to 0.12 Hz.

### Mean fractional amplitude of low-frequency fluctuations (mfALFF)

Amplitude of Low-Frequency Fluctuations (ALFF) quantifies the intensity of low-frequency fluctuations in the BOLD signal across the whole brain. It reflects the strength of the spontaneous neural activity at those low frequencies^16^. As mentioned above, we removed the linear trend of preprocessing data and selected temporal bandpass filtering (0.01–0.12 Hz). We conducted the fALFF analysis^74^ with the following steps. First, we applied a fast Fourier transform (FFT) to transform the time course of each voxel (time domain) into the frequency domain, and the power spectrum was then acquired. Second, we calculated a given frequency in the power spectrum to the square of the amplitude of the frequency; each frequency of the power spectrum was calculated as a square root. Third, the fALFF was defined as the averaged square root at each voxel. Finally, the fALFF value was divided by the mean fALFF (mfALFF) value for standardization.

### Mean regional homogeneity (mReHo)

Regional Homogeneity (ReHo) measures the local synchronization of BOLD signal fluctuations within a small cluster of neighboring voxels within the brain. It assesses how similar the time series of BOLD signals are among adjacent voxels in a given region, indicating regional functional connectivity^17^. We performed bandpass filtering (0.01–0.12 Hz) on the preprocessed images to measure ReHo. Based on the voxel-by-voxel in the ReHo analysis, the time course of each voxel was calculated using Kendall’s coefficient of concordance (KCC)^17^. By calculating the KCC of a given voxel, a KCC was assigned to this voxel and those of its nearby 26 neighbors. The KCC program was performed on REST in MATLAB. We generated an individual ReHo map for each dataset. In addition, the mean ReHo (mReHo) value was defined as the KCC value divided by the average KCC value. The mReHo value is assigned to each voxel, within 0 and 1. The cluster indicated higher concordance or local homogeneity if the mReHo value was closer to 1.

To evaluate the comparison differences in mfALFF/mReHo, we conducted a two-sample t-test in SPM12. To evaluate the association between the various cognitive tests and image indices, multiple regression analysis of the was performed in the 28 HTN participants. Sex, age, and years of education were considered covariates to avoid the original difference. A false discovery rate (FDR)-corrected *p* value of < 0.05 was considered statistically significant.

### Graph theoretical analysis (GTA)

First, each resting-state functional image was acquired and then transformed into MNI space. We then computed this transformation matrix in MNI space using the transformation matrices obtained from the two registers described in the procedure above. The functional images were spatially normalized to an automatic anatomical labeling (AAL) template of the underlying MNI space. We segmented the whole brain of each subject into 90 regions based on the AAL atlas^75^, with each region considered a node^76,77^. The connection between each node was viewed as an edge. The 90 × 90 connectivity matrix was then computed through Pearson correlations for each participant. After functional connectivity analysis, connectivity matrices were obtained from the Functional Connectivity Toolbox (CONN, Neuroimaging Informatics Tools and Resources Clearinghouse, NITRC). Finally, we performed a graph theory analysis using this connection matrix.

The Graph Analysis Toolkit (GAT, Stanford University School of Medicine, Stanford, CA, USA) was applied to calculate topological parameters and analyze functional connectivity networks^78^. A minimum network density of 0.2 was selected if the individual networks were not fragmented. The maximum network density was set at 0.6, and the threshold depended on the percentage of connections remaining. In summary, we extracted two groups’ networks with different correlation thresholds ranging from 0.2 to 0.6 in steps of 0.01.

The topological parameters of complex brain networks are transitivity, assortativity, modularity, clustering coefficient (C), normalized clustering coefficient (γ), local efficiency (E_local_), characteristic path length (L), normalized characteristic path length (λ), global efficiency (E_global_), and small-worldness (σ)^78^. We manually selected the area under the curve (AUC) between 0.2 and 0.6 in the density range and performed a two-sample t-test to find statistically significant differences between the two groups in these topological parameters. Density displays the current percentage of the number of edges for all possible connections.

### Network-based statistics (NBS)

Network-based statistics (NBS, Melbourne Neuropsychiatry Centre, The Melbourne University, Melbourne Health, Australia) attempts to determine the appropriate threshold connectivity to identify the underlying connectivity^20^. In this study, we used NBS to identify significant changes in all subnets connected in the group of hypertension-altered connections. A default threshold was calculated for each pairwise association to construct a set of supra-threshold links. In our experiments, we used nonparametric permutations (here, 5000 permutations) to obtain a distribution of edge numbers to assess the importance of each connected edge.

### Supplementary Information

Supplementary Tables.

## Data Availability

The data that support the findings of this study are available from the corresponding author upon reasonable request.
