# Supplementary material for: Investigating the effect of hypertension on vascular cognitive impairment by using the resting-state functional connectome
Source: Sci Rep. 2024 Feb 25;14:4580. doi: 10.1038/s41598-024-54996-9 (PMC10894879; doi:10.1038/s41598-024-54996-9)
Supplement: Supplementary file 1 — Supplementary Tables. [file 41598_2024_54996_MOESM1_ESM.docx]

**Table S1** Two-sample t-test results of mfALFF

| Region | side | MNI coordinates (mm) | | | p-value | clusters  (voxels) |
| --- | --- | --- | --- | --- | --- | --- |
|  |  | x | y | z |  |  |
| HTN > HC |  |  |  |  |  |  |
| Superior parietal lobule | L | -36 | -67 | 44 | < 0.02 | > 50 |
| Medial frontal gyrus | L | -12 | 53 | 4 | < 0.02 | > 50 |
| Inferior frontal gyrus | R | 27 | 26 | -19 | < 0.02 | > 50 |
| HTN < HC |  |  |  |  |  |  |
| Posterior cingulate gyrus | R | 3 | -56 | 5 | < 0.02 | > 100 |
| Superior temporal gyrus | L | -47 | 8 | 20 | < 0.02 | > 100 |
| Superior temporal gyrus | R | 45 | 9 | -17 | < 0.02 | > 100 |

All coordinates are given in Montreal Neurological Institute (MNI) space.

**Table S2** Two-sample t-test results of mReHo

| Region | side | MNI coordinates (mm) | | | p-value | clusters  (voxel) |
| --- | --- | --- | --- | --- | --- | --- |
|  |  | x | y | z |  |  |
| HTN > HC |  |  |  |  |  |  |
| Superior parietal lobule | L | -6 | -67 | 56 | < 0.02 | > 100 |
| Postcentral gyrus | R | 6 | -46 | 68 | < 0.02 | > 100 |
| Inferior frontal gyrus | R | 21 | 23 | -16 | < 0.02 | > 100 |
| HTN < HC |  |  |  |  |  |  |
| Precentral gyrus | L | -45 | -19 | 41 | < 0.02 | > 50 |
| Insula | L | -42 | -13 | 17 | < 0.02 | > 50 |
| Caudate | L | -9 | 14 | 11 | < 0.02 | > 50 |
| Fusiform gyrus | L | -39 | -9 | -29 | < 0.02 | > 50 |

All coordinates are given in Montreal Neurological Institute (MNI) space.

**Table S3** Summary of the correlation between CVVLT and mfALFF/ mReHo in the HTN group.

| Region | side | MNI coordinates (mm) | | | p-value | clusters  (voxel) |
| --- | --- | --- | --- | --- | --- | --- |
|  |  | x | y | z |  |  |
| Positive correlation (mfALFF) |  |  |  |  |  |  |
| Superior frontal gyrus | R | 29 | 65 | 3 | < 0.02 | >40 |
| Precentral gyrus | L | -54 | -7 | 32 | < 0.02 | >40 |
| Postcentral gyrus | R | 51 | -16 | 50 | < 0.02 | >40 |
| Positive correlation (mReHo) |  |  |  |  |  |  |
| Medial frontal gyrus | L | -9 | 50 | -1 | < 0.02 | > 50 |
| Inferior frontal gyrus | L | -53 | 26 | -5 | < 0.02 | > 50 |

All coordinates are given in Montreal Neurological Institute (MNI) space.

**Table S4** Summary of the correlation between TMT-A and mfALFF/ mReHo in the HTN group

| Region | side | MNI coordinates (mm) | | | p-value | clusters  (voxel) |
| --- | --- | --- | --- | --- | --- | --- |
|  |  | x | y | z |  |  |
| Positive correlation (mfALFF) |  |  |  |  |  |  |
| Inferior frontal gyrus | L | 27 | 19 | -11 | < 0.02 | > 60 |
| Negative correlation (mfALFF) |  |  |  |  |  |  |
| Middle frontal gyrus | L | -21 | 14 | 62 | < 0.02 | > 60 |
| Superior frontal gyrus | R | 15 | 26 | 62 | < 0.02 | > 60 |
| Positive correlation (mReHo) |  |  |  |  |  |  |
| Middle frontal gyrus | L | -30 | 35 | 47 | < 0.04 | > 50 |
| Superior frontal gyrus | L | -10 | 13 | 65 | < 0.04 | > 50 |

All coordinates are given in Montreal Neurological Institute (MNI) space.

**Table S5** Summary of the correlation between CGUOFT and mfALFF/ mReHo in the HTN group.

| Region | side | MNI coordinates (mm) | | | p-value | clusters  (voxel) |
| --- | --- | --- | --- | --- | --- | --- |
|  |  | x | y | z |  |  |
| Positive correlation (mfALFF) |  |  |  |  |  |  |
| Superior temporal gyrus | R | 64 | -29 | 8 | < 0.02 | > 50 |
| Inferior temporal gyrus | L | -55 | -46 | -15 | < 0.02 | > 50 |
| Positive correlation (mReHo) |  |  |  |  |  |  |
| Insula | L | -42 | -24 | -1 | < 0.02 | > 50 |
| Middle frontal gyrus | L | -46 | 20 | 29 | < 0.02 | > 50 |
| Inferior temporal gyrus | R | 40 | 0 | -42 | < 0.02 | > 50 |

All coordinates are given in Montreal Neurological Institute (MNI) space.

**Table S6** Summary of the correlation between DSS and mfALFF/ mReHo in the HTN group

| Region | side | MNI coordinates (mm) | | | p-value | clusters  (voxel) |
| --- | --- | --- | --- | --- | --- | --- |
|  |  | x | y | z |  |  |
| Positive correlation (mfALFF) |  |  |  |  |  |  |
| Superior frontal gyrus | L | -21 | -29 | 74 | < 0.02 | > 60 |
| Superior frontal gyrus | R | 9 | 2 | 74 | < 0.02 | > 60 |
| Middle frontal gyrus | R | 45 | -1 | 58 | < 0.02 | > 60 |
| Positive correlation (mReHo) |  |  |  |  |  |  |
| Superior frontal gyrus | L | -24 | 44 | 29 | < 0.02 | > 60 |
| Medial frontal gyrus | R | 4 | 52 | 9 | < 0.02 | > 60 |
| Insula | R | 36 | 20 | 5 | < 0.02 | > 60 |
| Precentral gyrus | R | 42 | -13 | 62 | < 0.02 | > 60 |

All coordinates are given in Montreal Neurological Institute (MNI) space.

**Table S7** Summary of the correlation between duration of hypertension and mfALFF/ mReHo in the HTN group

| Region | side | MNI coordinates (mm) | | | p-value | clusters  (voxel) |
| --- | --- | --- | --- | --- | --- | --- |
|  |  | x | y | z |  |  |
| Positive correlation (mfALFF) |  |  |  |  |  |  |
| Inferior frontal gyrus | L | -57 | 5 | 29 | < 0.02 | > 50 |
| Medial frontal gyrus | R | -6 | 29 | 46 | < 0.02 | > 50 |
| Positive correlation (mReHo) |  |  |  |  |  |  |
| Inferior frontal gyrus | L | -39 | 22 | 0 | < 0.02 | > 50 |
| Postcentral gyrus | R | 51 | -19 | 44 | < 0.02 | > 50 |

All coordinates are given in Montreal Neurological Institute (MNI) space.
